# Favorable Response to Repetitive Transcranial Magnetic Stimulation in a Refractory Fibromyalgia Case: Decrease in Pain Threshold After 20 Sessions

**DOI:** 10.1192/j.eurpsy.2026.11749

**Published:** 2026-07-31

**Authors:** A. Romero Teruel, C. Molina Liétor, P. Vázquez Giraldo, M. Leonor del Pozo

**Affiliations:** 1 Dr. Rodríguez Lafora Hospital; 2 Gregorio Marañón University General Hospital; 3 La Paz University Hospital, Madrid, Spain

## Abstract

**Introduction:**

Fibromyalgia is a chronic widespread pain syndrome often associated with central sensitization, and many patients remain refractory to conventional pharmacologic and non-pharmacologic therapies. Repetitive transcranial magnetic stimulation (rTMS) has been explored as a neuromodulation approach to modulate pain networks, especially via stimulation of the primary motor cortex (M1) or dorsolateral prefrontal cortex (DLPFC).

Some randomized controlled trials and long-term observational studies have reported sustained analgesic effects of rTMS in fibromyalgia and other chronic pain conditions. The mechanisms are thought to involve modulation of cortical excitability, enhancement of intracortical inhibition, and downstream effects on thalamic and subcortical pain pathways.

However, heterogeneity in stimulation parameters, target sites, and patient phenotypes has limited consensus on optimal protocols and predictors of response.

**Objectives:**

To present a clinical case illustrating a meaningful analgesic response—with reduction in pain threshold—after a full cycle of 20 rTMS sessions in a patient with refractory fibromyalgia, and to situate it in the context of existing evidence.

**Methods:**

**Patient Profile:** 57-year-old woman with long-standing fibromyalgia refractory to analgesics (including duloxetine 120 mg/d, gabapentin 900 mg/d, and conventional therapies), comorbid mood disorder (in partial remission), and functional impairment with gait difficulty and fatigue.

**Intervention:** rTMS delivered per standard depression protocol over left dorsolateral prefrontal cortex (20 sessions total) starting 11 June 2025. The treatment was well tolerated without significant adverse events.

**Outcome Measures:** Pain assessed by Visual Analog Scale (VAS/EVA) at baseline, mid-treatment, and end of treatment. Patient also reported subjective changes in mood, quality of life, and functional capacity.

**Results:**

Baseline VAS: 7-8/10

Midpoint (≈ session 10): VAS ~6/10

End of 20 sessions: further subjective improvement in pain (patient report) and mood

The patient described a perceptible increase in her pain threshold (i.e. lesser sensitivity to painful stimuli), enabling modest functional gains.

No serious side effects or dropouts occurred.

Based on this favorable response, a maintenance cycle was scheduled for September 2025.

**Conclusions:**

In this refractory fibromyalgia patient, a 20-session course of rTMS was associated with a clinically meaningful decrease in pain, increase in pain threshold, and subjective improvements in mood and function. This favorable response adds to the accumulating evidence supporting rTMS as a potential neuromodulatory tool in pain syndromes. Controlled trials with standardized protocols and long-term follow-up are needed to confirm efficacy, refine stimulation parameters, and identify predictors of response.

**Disclosure of Interest:**

None Declared

